# Can Yeast (*S. cerevisiae*) Metabolic Volatiles Provide Polymorphic Signaling?

**DOI:** 10.1371/journal.pone.0070219

**Published:** 2013-08-19

**Authors:** J. Roman Arguello, Carolina Sellanes, Yann Ru Lou, Robert A. Raguso

**Affiliations:** 1 Department of Molecular Biology and Genetics, Cornell University, Ithaca, New York, United States of America; 2 Laboratiro de Ecología Química, Facultad de Química, Universidad de la Repúlica, Montevideo, Uruguay; 3 Department of Plant Biology, Cornell University, Ithaca, New York, United States of America; 4 Department of Neurobiology and Behavior, Cornell University, Ithaca, New York, United States of America; University of Toronto, Canada

## Abstract

Chemical signaling between organisms is a ubiquitous and evolutionarily dynamic process that helps to ensure mate recognition, location of nutrients, avoidance of toxins, and social cooperation. Evolutionary changes in chemical communication systems progress through natural variation within the organism generating the signal as well as the responding individuals. A promising yet poorly understood system with which to probe the importance of this variation exists between *D. melanogaster* and *S. cerevisiae*. *D. melanogaster* relies on yeast for nutrients, while also serving as a vector for yeast cell dispersal. Both are outstanding genetic and genomic models, with *Drosophila* also serving as a preeminent model for sensory neurobiology. To help develop these two genetic models as an ecological model, we have tested if - and to what extent - *S. cerevisiae* is capable of producing polymorphic signaling through variation in metabolic volatiles. We have carried out a chemical phenotyping experiment for 14 diverse accessions within a common garden random block design. Leveraging genomic sequences for 11 of the accessions, we ensured a genetically broad sample and tested for phylogenetic signal arising from phenotypic dataset. Our results demonstrate that significant quantitative differences for volatile blends do exist among *S. cerevisiae* accessions. Of particular ecological relevance, the compounds driving the blend differences (acetoin, 2-phenyl ethanol and 3-methyl-1-butanol) are known ligands for *D. melanogaster*s chemosensory receptors, and are related to sensory behaviors. Though unable to correlate the genetic and volatile measurements, our data point clear ways forward for behavioral assays aimed at understanding the implications of this variation.

## Introduction

A requisite condition for evolutionary change is heritable variation segregating within a population. In order to understand the forces governing the change, evolutionary biology is preoccupied with quantifying patterns of variation in order to measure and test for the effects of selection. Evolutionary biology has a rich history describing organismal phenotypic and genetic diversity over varying geographies and physical distances, and increasingly sophisticated methods to infer the evolutionary processes that have led to patterns of diversity continue to develop rapidly [1]–[9]. Traditionally, genetic variation has been treated in isolation from the extensive web of ecological connections within which the organism is embedded. However, there has been growing attention, as well as increased technological feasibility, to combine genetic variation with ecologically relevant phenotypic data. This effort has been referenced by terms such as “community and ecosystem genetics” or “landscape genetics”, and aims to provide a more detailed understanding of how a population or species affects, or is affected by, the larger ecological network to which it belongs [10]–[12].

A particular area of research that holds remarkable promise towards bridging ecology and genetics is the study of chemical signaling between hosts and their visitors. Host-visitor systems occur ubiquitously, and many of the best studied systems involve insects and their hosts (most often plants) [13]–[17]. These systems benefit from their suitability for detailed lab and field-based behavioral studies, and are amenable to precise characterization of the chemical makeup of the cues that elicit the behavioral response(s) that mediate their interactions (e.g. oviposition or proboscis extention). An outstanding example of such a system is the apple (*Malus pumila*)-infesting and hawthorn (*Crataegus* spp) flies of the *Rhagoletis pomonella* (Diptera; Tephritidae) species group. In the mid 1800s it was noted that a N. American *Rhagoletis* population experienced a host shift away from the native hawthorn host towards introduced apple trees [18], and it was subsequently suggested that the shift had occurred sympatrically [19]. Through a combination of chemical, behavioral, and electrophysiological studies, it has been demonstrated that differences in chemical cues emitted from the fruits of these trees have significantly contributed to the host shift [20], [21].

Additional layers of community interactions can be considered through the recognition of the micro-organisms that inhabit the fruits, flowers, and surfaces of host plants. By feeding on, or from, these substrates, insects often serve as dispersal agents for the micro-organisms. Intriguingly, because these micro-organisms often produce their own volatile compounds, or can induce a plant to emit variations in its own volatiles [22], [23], it is an open question to what extent host plant signals involve the chemical (or physical) contribution of the microbes they carry. With respect to microbe-plant-insect interactions, *Drosophila* systems have made notable contributions. There have been ongoing efforts to understand host-visitor relations among *Drosophila* flies and yeasts dating back to the mid 20th century [24]–[30]. It has long been established that *Drosophila* larvae rely on yeasts for nutritional contributions [31] as well as for their role in aiding to detoxifying otherwise toxic plant material that the flies ingest [32]. In addition, adult *Drosophila* have been shown to be important vectors for yeast cells, assisting in the dispersal and outbreeding rate of this microbial fungus [33]. Evidence for this proximal relationship between *Drosophila* and yeasts was recently strengthened through an experimental study demonstrating that *Drosophila melanogaster*, which is attracted to – and mates upon – decaying fruit, is primarily drawn to the fruit through the volatiles generated by the yeast (*Saccharomyces cerevisiae*) fermenting upon them [34].

The *Drosophila*-yeast pair is a particularly promising model system for investigating ecological interaction and coadaptation, due both to the historical background on the relationships across multiple species of flies and yeast, and also because the specific species pair *D. melanogaster*-*S. cerevisiae* present two of the most powerful molecular genetic and genomic models available. In particular, efforts to investigate the sensory preferences of *D. melanogaster* in relation to its ecology should significantly benefit from the fact that *D. melanogaster* is one of the best understood systems regarding the molecular and neurological foundations of olfaction and gustation [35]–[43]. Moreover, both species have high quality genome assemblies available, with population genomic resequencing efforts continuously expanding [44]–[47]. These resources are providing a detailed understanding of subtle genetic differences across geographic localities, and are beginning to be related to higher-level phenotypes [46], [48].

A necessary first step in testing for population variability in the ecological interactions between *D. melanogaster* and *S. cerevisiae* (or between any other insect that interacts with *S. cerevisiae*) is to explore the potential for population variability in yeast signaling. To do this, we set up a series of common garden experiments using geographically and genetically distinct strains of *S. cerevisiae* from which we collected metabolic headspace volatiles for extensive chemical analyses. We reasoned that given the important contribution of yeast volatiles to fly-fruit interactions [39], the results of such a study should be informative to both present and future fly-yeast-fruit ecological studies, and should help to further develop these model organisms as a powerful “ecological genetics” system-pair.

Recently, several *S. cerevisiae* whole genome resequencing efforts were completed in which accessions from around the world, isolated from diverse substrates, were sampled [45], [47]. Population genetic analyses demonstrated considerable population structure (up to 5 genetic groupings), especially between strains domesticated for distinct fermenting purposes (sake, wine) and strains used in scientific laboratories. In parallel to the genomic analyses, Liti et al. (2009) also carried out extensive phenotypic measurements related to growth. Surprisingly, they found that clustering of these phenotypes could qualitatively recapitulate the topology of the genetic clusters, and that the most discrete phenotypic separation existed for rapid growth between the wine, European, and mosaic (admixed) strains and the remaining North American, Malaysian, and African strains [45]. Drawing from these studies, we predicted that if we subsampled from the diverse genetic clusters observed in these previous studies, we would be able to capture variation in volatile chemical composition across this species if it exists. In addition, although our sample size is necessarily smaller than Liti et al.'s, the data would allow for tests of correlations between the volatile clusters and genetic clusters. Our results revealed considerable plasticity in metabolic volatiles within strains, but still we observed significant differences between several strains of *S. cerevisiae*, and were able to attribute most of the between-accession variation to particular compounds. Intriguingly, most of the differentiated compounds are known ligands for *D. melanogaster* chemosensory receptors, and have been shown to be related to sensory behavior. The genomic sequence analyses indicated that our volatile phenotyping spans a broad genetic panel, as supported by comparable genetic clusters in our study and those in the larger population surverys [45], [47]. However, attempts to identify correlations between the volatile data and genomic data were unsuccessful, most likely due to our relatively small sample sizes and the inherent noise of volatile sampling. We conclude with considerations over the implications of our common garden design on estimating variation in metabolic volatiles, and on the general need to improve methodological approaches and throughput aimed at addressing population structure in the “invisible phenotypes” of interest to chemical ecologists.

## Results

### Sampled Strains Are From Distinct Genetic Clusters

**Figure 1:**
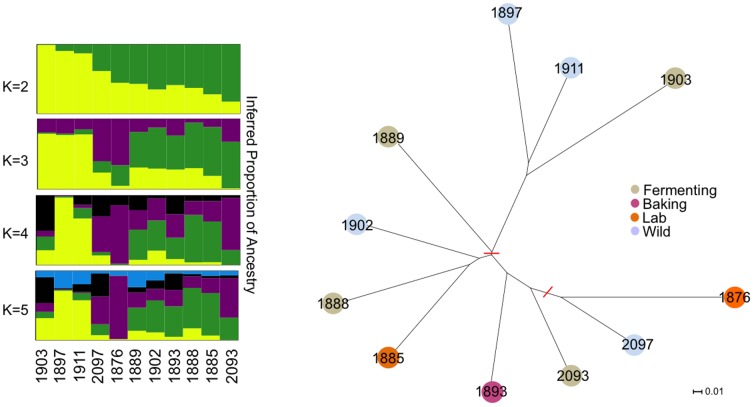
Genetic relationships between the 11 yeast accessions for which genomic sequence is available. Left: Inferred proportion of ancestry estimated for 2–5 genetic clusters. Right: A Neighbor Joining tree for the same yeast accessions. All branches have bootstrap values greater than 95% except for the two marked with red lines (upper branch = 55.8, lower branch = 74.9). Color-coding on tree tips indicate the grouping of the strains according to Table 1.

Based on a subset of overlapping DNA sequences collected from previous genome sequencing projects (Table 1; see Methods), the Neighbor Joining Tree (Fig. 1) shows a long branch separating the two Malaysian strains (1911, 1897) and the single Sake strain (1903), and is in general agreement the branch placement previously observed [45] (see their Fig. 1C). The addition of the two samples from the study of Schacherer et al. (2009) make no impact on Malaysian/Sake-split, but does alter the tree topology by creating a well supported additional clade that includes reference lab strain 1876 and the baking strain 1893. The remaining 4 strains form a third clade with weaker support for the branch placement, consistent with the pattern seen for these mosaic strains along the long branch between the Wine/European strains and North American strains [45]. Analyses of structure results indicate that the samples in our study contain varying proportions of shared ancestry. Prior probabilities computed from the three runs with K = 1∶6 generally increase from K = 1 to K = 5 and then quickly drop off at K = 6. The highest prior for each run was with K = 5 (Fig. S1).

**Table 1:** Summary information for the 14 yeast accessions used in this study.

Strain	OS	Location	Source	Group	Genomic Data	NJ Tree Color Coding
YS2	1893	Australia	baker strain	baking	Liti et al.	red
Y12	1903	Africa	palm wine strain	fermenting	Liti et al.	green
DBVPG6040	1889	Netherlands	fermenting fruit juice	fermenting	Liti et al.	green
YIIc17.E5	1888	Sauternes, France	wine	fermenting	Liti et al.	green
S288c	1876	California	rotting fig	lab	Liti et al.	orange
UWOPS83.787.3	1911	Bahamas	Fruit, opuntia stricta	wild	Liti et al.	blue
UWOPS03.461.4	1897	Malysia	Nectar, Bertam palm	wild	Liti et al.	blue
273614N	1885	RVI, Newcastle	clinical isolate (fecal)	lab	Liti et al.	orange
DBVPG1853	1902	Etheopia	White Teff	wild	Liti et al.	blue
YpS163	2097	USA	Oak exudates	wild	Liti et al.	blue
UC8	2093	South Africa	wine	fermenting	Schacherer et al.	green
SB (S. boulardii)	2094	Indonesia	lychee fruit	wild	N/A	N/A
UCD612	2100	Pennsylvania, US	oak exudate fruit	wild	N/A	N/A
Y4	2106	Indonesia	fruit	wild	N/A	N/A

### Yeast Volatile Analysis: non-polar GC-MS screen reveals strain-specific volatile ratios

Thirty two volatile compounds were identified from yeast headspace using the non-polar GC column. The identities and relative abundances of these compounds, and their relative contributions to the total volatile blend of each yeast accession, are summarized in Table S1. Volatile compounds common to all samples included the dominant component 3-methyl butanol and a number of short-chain alcohols, acids and esters. Twelve additional yeast volatile compounds resulted from the apparent esterification of ethanol, 3-methyl butanol and 2-phenylethanol – three of the most abundant alcohols present in yeast headspace – with different organic acids present in grape juice (e.g. hexanoic, octanoic and decanoic acid). Finally, we detected low amounts of two unique compounds without obvious biosynthetic affinity to the more typical fermentation volatiles, including nerolidol (a sesquiterpene alcohol) and dihydro-2-methyl-thiophenone (an S-bearing heterocyclic compound).

Principal Component Analysis (PCA) of the 8 most consistent volatiles identified by TD-GC-MS on the non-polar GC column resulted in three factors (PCs) with eigenvalues greater than unity, explaining roughly 75% of the variance in the data set (Table 2). Component score coefficients indicated unambiguous loading relationships between PC 1 and ethyl esters, PC 2 and 2-phenylethanol, 3-methyl butanol and 3-methyl butyl acetate, and PC 3 and acetoin and isobutyric acid (Table 2). Examination of the factor loadings for all samples identified some accessions with high loadings on one factor (2097 for PC1, 1885 for PC2, 1893 for PC3), others with distinctive loading patterns (1888 low for PC1 and PC2 but high for PC3; 2094 high for PC1 but low for PC2; 2106 low for PC1 but high for PC2), and one (1876) with especially low loadings for PC2 (Table 2). Based on their distinctive loading patterns, these seven accessions were chosen for the second round of analyses on the polar GC column (see below).

**Table 2:** Principle component analyses and loading summaries for yeast volatile data.

	A	B			C
Factor	1	2	3	1	2	3	1	2	3
Eigenvalue	3.029	1.753	1.245	3.005	2.498	1.047	3.496	1.531	1.397
% Variance Explained	37.86	21.91	15.56	37.56	31.22	13.09	43.7	19.14	17.46
Cumulative			75.34			81.87			80.3
acetoin	0.114	−0.017	0.639	0.177	0.038	0.731	−0.128	0.081	0.584
3-methyl butanol	0.073	0.361	0.111	0.11	0.394	0.121	−0.017	0.37	0.019
isobutyric acid	−0.081	−0.053	0.496	−0.008	0.038	0.468	−0.215	0.46	0.066
3-methyl butyl acetate	−0.082	0.441	−0.224	−0.057	0.343	−0.114	0.081	0.322	−0.163
ethyl hexanoate	0.37	−0.036	0.056	0.405	0.038	0.174	0.366	0.014	−0.164
2-phenylethanol	−0.155	0.444	0.037	−0.023	0.369	0.079	0.054	−0.11	0.503
ethyl octanoate	0.348	0.006	0.032	0.391	0.036	0.082	0.405	−0.103	−0.002
ethyl decanoate	0.333	−0.17	−0.015	0.281	−0.057	−0.05	0.3	−0.137	0.204

A: Full data set, 14 accessions, non-polar column.

B: Subset of data, 7 accessions chosen for follow-up, non-polar column.

C: Follow-up study, 7 accessions, polar column.

**Figure 2:**
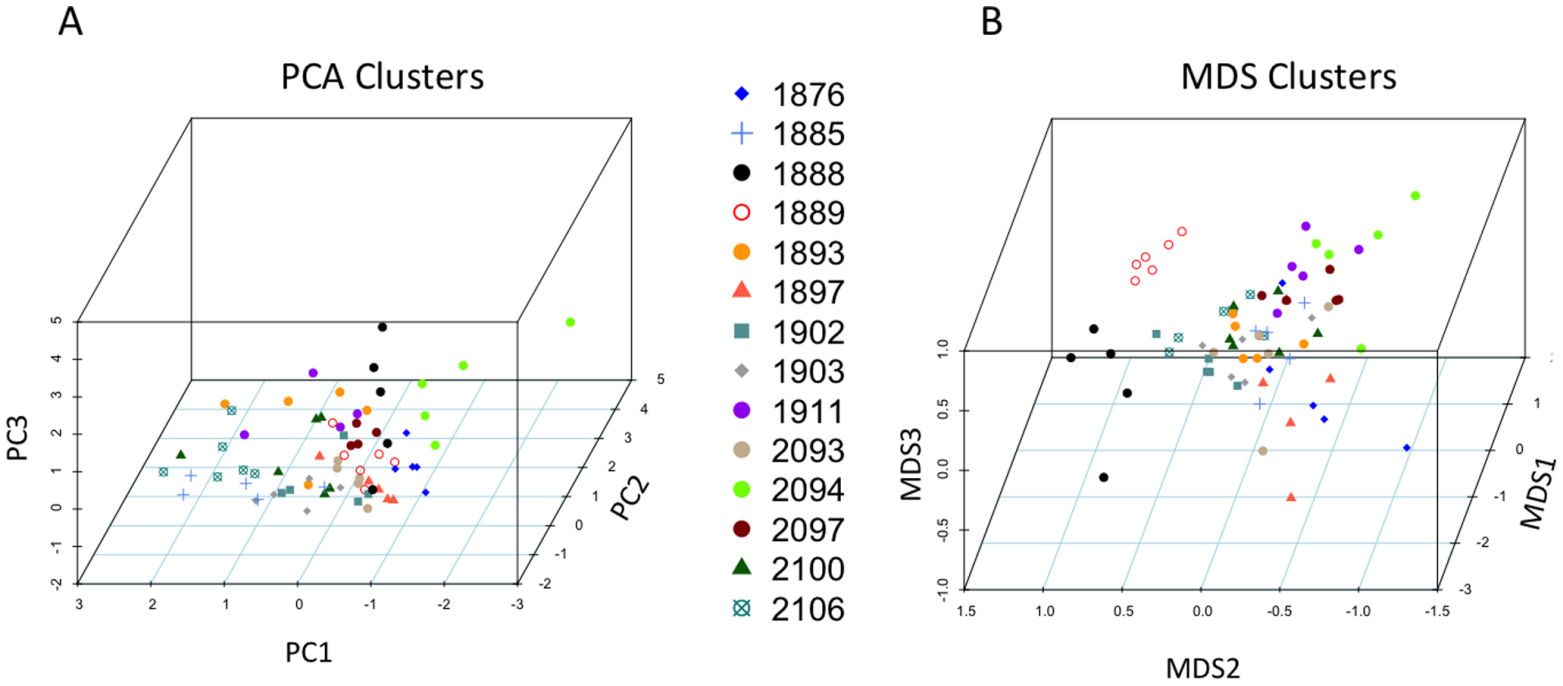
Summaries for the ordination of non-polar GC-MS volatile data for all 14 yeast accessions (see legend inset). A) Principal Components Analysis (PCA) on the 8 core volatiles, showing the compounds that loaded most highly on the first three significant factors (PC1-3). B) Multidimensional Scaling (MDS) of all 32 volatiles, showing the compounds that frequently explained differences between the accessions showing greatest differences along specific axes (MD1-3) in Cartesian scent space (e.g. accessions 1888 vs. 2094 along MDS2, explained largely by acetoin and isobutyric acid); see also Table S2. Note the similarity between compounds annotated with MDS axes in panel B and PC factors in panel A.

Multidimensional Scaling (MDS) analysis was performed on the full blend of 32 volatile compounds identified using TD-GC-MS on the non-polar GC column. Further exploration of differences between yeast strains was justified because ordination of the Bray-Curtis index produced a significantly structured data set (ANOSIM global R = 0.515, p = 0.0001) with relatively low stress (3-D = 0.09; 2-D = 0.13). The three dimensional MDS ordination of the Bray-Curtis index generated by the full volatile blend is shown in Fig. 2 (panel B). Although the replicates of several strains show overlapping distributions in MDS, some strains show clear separation along specific MDS axes. SIMPER analysis was used to explore which specific volatiles might be associated with these axes. SIMPER indicated high average similarity in scent composition within strains (grand mean = 84.29%). Comparisons between strains using SIMPER revealed that quantitative differences between compounds common to all strains (including the 8 compounds used in PCA) were largely responsible for distributional differences in MDS, and that topological differences between strains in volatile phenotype space are largely explained by the compounds that loaded most strongly on PC1, 2 and 3 (see Table 2). Specifically, there were no low-abundance or unique volatiles that contributed to the upper 60% of cumulative differences between strains; these differences were nearly always driven by 3-methyl butanol, the dominant yeast volatile in our analyses, as is summarized in Table S2. Strains that differed primarily along MD1, including the fermenting strains 1888 (YIIc17.ES from Sauternes, France) and 1889 (DBVPG6040 from the Netherlands) did so largely due to different amounts of isobutyric acid and acetoin (− MD1 values; see PC3 in Table 2). In contrast, strains that differed primarily along MD2, such as 1889 and wild strain 1911 (UWOPS83.787.3, from cactus fruit in the Bahamas) partitioned greater emissions of ethyl esters (+ MD2 values; see PC1 in Table 2) vs. 3-methyl butanol and 3-methyl butyl acetate (− MD2 values; see PC2 in Table 2). Finally, strains that differed primarily along MD3, such as strains 1911 and 1885 (273614N, a clinical fecal isolate from the UK) were typified by marked differences in emissions of 2-phenylethanol (- values).

### Yeast Volatile Analysis: follow-up polar GC-MS screen resolves volatile alcohols, but increases noise

Thirty six total volatiles were identified from TD-GC-MS analysis of strains 1876, 1885, 1888, 1893, 2094, 2097, 2106 (see Table S1). All compounds identified from the previous analysis were resolved on the polar GC column except for the S volatile dihydro-2-methyl thiophenone. Eight small volatiles that were either not detected or not well resolved by the non-polar GC column were consistently detected using the polar column, including ethanol, ethyl acetate, propanol, isobutanol, butanol and pentanol (Table S2). Isobutanol was the most abundant of these compounds, constituting from 11 to 34% of total emissions on average. Otherwise, 3-methyl butanol (combined in these analyses with small amounts of 2-methyl butanol) remained the dominant scent component, accounting for 50–70% of total emissions from different strains. Two yeast volatiles known to attract *Drosophila* flies – acetic acid and acetoin – were consistently well-resolved in all replicate samples using the polar column. However, ethanol and ethyl acetate eluted during the isothermal phase of the GC temperature program and had large, square peaks that we did not integrate due to their shapes.

Principal Component Analysis (PCA) of the same 8 volatiles on the polar GC column resulted in PCs with eigenvalues greater than unity, explaining roughly 80% of the variance in the data set (Table 2). Component score coefficients indicated the same loading relationships between PC 1 and ethyl esters, but this time 3-methyl butanol, its acetate and isobutyric acid loaded positively on PC2, whereas 2-phenylethanol and acetoin loaded positively on PC3 (Table 2). Surprisingly, the factor loadings for all samples revealed inconsistent results between the two analyses, in terms of patterns associated with specific yeast strains. For example, ethyl esters were high in strains 2094 and 2097 and low in strains 1888 and 1885 in the nonpolar column analysis, but were high in strains 2097 and 1885 and low in strains 1888 and 2094 in the polar column analysis. Strain 2106 had been high for 2PE in the original analysis (Table 2), but was low in the subsequent analysis, whereas the converse pattern was observed for strain 1876. When PCA was restricted to the four volatiles from the behaviorally active blend (acetoin, acetic acid, 3-methylbutanol, 2-phenylethanol) identified by Becher et al. (2012), only one PC factor was identified with an eigenvalue greater than unity, explaining 54% of total variance.

MDS was performed on all VOCs from the 7 yeast strains. The Bray-Curtis similarity index produced from these data was analyzed using ANOSIM as before, revealing a global R = 0.117, p = 0.022, suggesting less strain-specific structure than was observed with the full 14 strains. Accordingly, the 2-D MDS (Strain 0.08) plot revealed no obvious clusters by strain, and SIMPER showed that average similarity within strains was substantially lower in this analysis (grand mean = 66.13%) than was observed in the initial analysis (grand mean = 84.29%). Thus, further exploration of differences between strains was not justified.

Instead, we explored the sources of high variance within strains by considering the biosynthetic origins of certain compounds. We used linear regression on ln-transformed, normalized, summed peak areas for esters derived from 2-phenylethanol and 3-methylbutanol as dependent variables, with the abundance of their respective alcohols as independent variables. The pooled abundance of all 2-phenethyl esters was significantly correlated with the amount of 2-phenylethanol (R^2^ = 0.515, F = 44.61, p>0.001), and similarly, the total amount of all isoamyl esters was significantly correlated with the amount of 3-methylbutanol (R^2^ = 0.337, F = 21.35, p>0.001; see discussion).

### No Correlation Between Genetic Clustering and Volatile Clustering

Despite observing structure in our volatile data that separates particular yeast accessions in our MDS and PC plots, we did not observe significant correlations between distance matrices generated from these data and the 11/14 strains for which we have genomic SNP data (Centroid-based Mantel Test p-value = 0.58; Mantel Tests over replicates p-value>0.5). The non-significant Mantel test results are qualitatively consistent with the conflicting genetic trees generated by hierarchically clustering and the trees generated by volatile-genetic distance matrices (data not shown). Similarly, we did not observe statistically significant improvements in predictive power with any of our models using the Structure-defined clusters (K = 3) used as categorical response variables or when using the continuous proportion of ancestry matrix for the K = 2 scenario (p-values>0.3).

## Discussion

Natural Selection acts on variation segregating within a population and can lead to geographical differences for fitness-related phenotypes. Chemical signaling represents a dynamic ecological trait that can vary over vastly different scales, and it is expected that natural variation in this phenotype carries significant evolutionary costs or benefits related to, for example, pollination and dispersal [49], [50]. Variation in chemical signaling can also impact the population dynamics of the organism(s) that target the signal(s). This can potentially result in changes in the modes of attraction and population subdivision [20], [51], [52]. Because they are amenable to experimentation, and due to their outstanding diversity, insect-plant systems have provided enormously productive research angles for understanding chemical signaling and its evolution [53].

Motivated by the importance of *D. melanogaster* as a model for sensory biology, and the recent clarification that yeast - another outstanding genetic model - provides a primary food source and emits highly attractive chemical signals targeted by flies [34], we set out to ask whether geographically diverse *S. cerevisiae* are capable of producing polymorphic signals, presumably through natural variation segregating at loci governing volatile biosynthesis. To our knowledge, this is the first exploration of population-level variation in yeast volatile emissions. As is clear from the provenance of the accessions studied here, *S. cerevisiae* is capable of colonizing a great diversity of substrates, from fruit to feces (see Table 1). Thus, in an effort to simplify potentially complex genotype x environment interactions, and to establish the extent to which metabolic volatile variation is genetically determined, we carried out our experiments under a common garden paradigm, on a standardized substrate (grape juice).

### Tapping into genetically variable model systems

By combining available genomic sequence data and scent chemistry, we were able to robustly access the genetic diversity of our sampling, and also to investigate the ability to uncover phylogenic signal available in the yeast's chemical signals. Our volatile analyses have provided quantification for each yeast's signal profile under the standardized growth conditions, and have allowed us to identify particular accessions as outliers for specific compounds. Encouragingly, nearly all volatile compounds underlying our observed between-accession differences are active ligands for known *D. melanogaster* sensory receptors, which, in turn, have been associated with sensory-related behaviors (Table 3).

**Table 3:** Relating olfactory receptors to significant compounds resulting from the PCA analysis.

PC 1			
Compound	OR receptors	OR Sensilla Classes	Citations for functional properties of receptors & *D. melanogaster* olfactory-related behavior
ethyl hexanoate	OR22a, OR7a†, OR10a†, OR35a, OR47a, OR47b†, OR67a, OR67c†, OR85a†, OR85b, OR98a†	ac1, ac2†, ac3, ac4	Richgels and Rollmann (2012); Silbering et al. (2011); Hallem et al. (2006)
ethyl acetate	OR42b, OR22a, OR 43b, OR47a, OR59b, OR85a	ac1†, ac2†, ac3, ac4†	Root et al. (2011); Silbering et al. (2011); Hallem et al. (2006)
ethyl benzoate	OR67a,OR98a, OR7a†, OR10a, OR23a†, OR35a†, OR43a†, OR49b†, OR67c†, OR85a†	ac1, ac2†, ac3, ac4†	Silbering et al. (2011); Hallem et al. (2006)
ethyl lactate	OR67c,OR9a, OR22a, OR43b, OR47b†, OR59b, OR85b, OR85f, OR98a	–	Hallem et al. (2006)
ethyl 3-hydroxybutyrate	OR85a, OR7a†, OR9a, OR10a, OR22a, OR35a, OR43b, aOR47b†, OR67a, OR67c, OR85b, OR85f, OR88a†, OR98a	ac1, ac2†, ac3, ac4†	Silbering et al. (2011); Hallem et al. (2006); Stensmyr et al. (2003)
PC 2			
Compound	OR receptors	OR Sensilla Classes	Citations for functional properties of receptors & *D. melanogaster* olfactory-related behavior
2-Phenylethanol	OR67a, OR10a, OR35a, OR49b, OR67a, OR98a†	ac1, ac2†, ac3†, ac4†	Becher et al. (2012); Silbering et al. (2011); Hallem et al. (2006); Zhu et al. (2003)
3-Methyl-1-butanol	OR7a, OR9a, OR1a9, OR22a, OR35a, OR43a, OR47b†, OR67a, OR67c, OR82a, OR85a†	ac1, ac2†, ac3†, ac4†	Becher et al. (2012); Silbering et al. (2011); Hallem et al. (2006)
3-methyl butyl acetate/isopentyl acetate	OR2a, OR7a†, OR9a, OR10a, OR19a, OR22a, OR43b, OR47a, OR47b†, OR67a, OR85a†, OR85b, OR98a	ac1†, ac2†, ac3, ac4†	Silbering et al. (2011); Hallem et al. (2006); Stensmyr et al. (2003)
PC 3			
Compound	OR receptors	OR Sensilla Classes	Citations for functional properties of receptors & *D. melanogaster* olfactory-related behavior
acetoin	OR92a, Or7a†, OR19a†, OR23a†, OR43a†, OR59b, OR67c†	ac1†, ac2, ac3, ac4†	Becher at a.l (2012); Silbering et al. (2011); Becher et al. (2010); Hallem et al. (2006); Stensmyr et al. (2003)
isobutyric acid	IR64a	ac1†, ac2, ac3, ac4	Silbering et al. (2011); Ai et al. (2010)

† inhibotory response.

We first determined the extent of genetic relatedness of the yeast accessions that we have phenotyped by utilizing the available genomic sequences for 11 of the 14 accessions [45], [47]. The results of our model-based inference of genetic clustering indicate that we sampled from a representatively diverse range of populations, despite having phenotyped a relatively small number of strains compared with the sample sizes in the two studies from which we mined the data. Analysis of Structure output suggests 2–5 clusters within our sample, with the highest posterior probability indicating five for the triplicate runs we executed (Fig. 1; Fig. S1). This finding is consistent with the 5 clusters reported previously [45], [47]. The topology of the neighbor joining tree also remains qualitatively consistent despite pooling across studies (Results). Overall, the genetic data indicate that the *S. cerevisiae* accessions that we have phenotyped are both geographically and genetically diverse, and that our chemical studies should not suffer from over-sampling closely related genotypes and should instead be informative as to the extent that their volatiles can vary across populations.

### Tracking the variance of invisible phenotypes

We made every effort to produce unbiased volatile data sets from these diverse strains, from the use of a standardized substrate in a common garden setting to the randomized block design of data collection over 21 days, and the double-blind manner in which data were coded and analyzed by our research team. We chose a highly sensitive, state-of-the-art direct thermal desorption (TD) GC-MS device to analyze yeast volatiles because we knew that many of the behaviorally important compounds with relation to *Drosophila* attraction are small, highly volatile molecules that would be masked by solvents (e.g. methylene chloride, hexane) in conventional solvent-eluted GC-MS sample analysis, and because SPME, a solvent-free alternative for GC-MS volatile analysis, is an equilibrium-based method that is ill-suited for rigorous quantitative analyses such as PCA [54].

TD-GC-MS generates headspace samples that are completely consumed during a single GC injection. It can be challenging to calibrate such samples (e.g. through the use of an internal standard), especially when volatiles are collected from a liquid matrix, as was the case in our study (grape juice). For that reason, we took especial care to standardize all aspects of headspace collection, including glassware volume, yeast densities and substrate concentrations, temperature and duration of incubation, vacuum pump flow rates and amount of Tenax sorbent in each volatile trapping filter. In addition, we chose methyl anthranilate, the character-bearing note of grape juice [55] and a compound that was not chemically modified by yeast fermentation, as the internal standard for our samples.

Our GC-MS analyses of scent chemistry using all 14 yeast accessions and a standard non-polar GC column revealed reproducible variation associated with several of the strains included in this study, as well as a data set full of compounds previously documented from yeast volatile analyses (Table 2) [34], [56]–[58]. When we extended our multivariate analyses to include the full data set of volatiles, we expected variable, low abundance compounds (e.g. 2-phenethyl esters) to contribute more structure to overall differences between strains in volatile phenotype space, especially given that such esters were strongly correlated with large amounts of their putative substrate, 2-phenylethanol (see Results). Instead, the compounds most responsible for clustering differences among strains in MDS (Fig. 2) were, by and large, the same compounds identified with strong loadings on the three significant PCA factors (see Table 2).

We repeated our common garden, randomized block headspace collection and TD-GC-MS analysis on additional replicates of the 7 most chemically divergent yeast strains with a polar GC column, in order to better resolve the small, highly volatile compounds (e.g. acetic acid, acetoin) known to function as fly attractants. To this end, we successfully and consistently resolved these compounds, plus 5 additional short chain alcohols too small to have been resolved on the non-polar GC column (Table S2). With this second data set in hand, we were curious as to whether the same strain-specific patterns identified from all 14 yeast accessions on the non-polar GC column would be observed using the polar column. Indeed, the same volatile compounds were found to load heavily on PC factors (Table 2), but with subtle differences from the previous analysis (Table 2). However, in this data set the significant quantitative variation associated with these volatiles was distributed within strains, and the previously observed clustering patterns were not conserved in either PCA or MDS analyses.

The inconsistencies between the polar and non-polar GC-MS data sets do not appear to be artifacts of sampling effort. When the non-polar GC-MS data are resampled including only the 7 yeast accessions used in the polar GC-MS analysis, the results are quite similar to those of the full 14 accession analysis, both in terms of specific compound loadings on PC1-3, and also in terms of the robustness of accession specific patterns (see Table 2). Nor do these inconsistencies appear to be artifacts of experimental date, as ANOSIM revealed a non-significant interaction between date and strain (R = −0.023, p = 0.54) for the polar column samples. Instead, these samples simply appear to be more variable across the experiment than those that were analyzed a year earlier on the non-polar GC column. The coefficients of variance (CV) were more than twice as large for peak normalized total odor (CV = 1.23) and methyl anthranilate peak areas (CV = 0.90) for the polar column samples (n = 37) as they were for non-polar column samples (n = 73), either for all 14 yeast accessions (total peak areas CV = 0.50, methyl anthranilate peak areas CV = 0.40) or for the same 7 accessions used in the repeated analyses (CVs are the same). Finally, only 3 of 73 non-polar samples showed evidence of excessive fermentation due to the presence of glycerol (and thus were omitted from further analysis), whereas 7 of 44 polar column samples were omitted due to the presence of large glycerol peaks. In summary, for reasons that remain unclear, our polar column analyses provided a more comprehensive view of the diversity of small volatile alcohols in yeast headspace, at the cost of greater non-biological variation between samples. Further protocol refinement will be necessary to better understand and eliminate the sources of experimental noise.

Nevertheless, the quality of our non-polar column data set is strongly borne out by two observations. First, we observed consistent clustering and high overall chemical similarity (cf. 84%) on average among replicates of most strains (Fig. 2). Second, the observed degree of differentiation actually exceeds that of population-level studies of geographic variation in floral volatiles conducted in our laboratory [52], [59].

### Methodological Considerations

Using a unique metabolomic approach to phenotype genetically defined yeast accessions, we were successful in quantifying volatile differences between accesssions. Unfortunately, these results were not consistently structured in a way that linked genetic and phenotypic volatile differences with each other. The emergence of significant clustering in scent space (Fig. 2) from data collected using a randomized block design in a common garden indicates that there are genetic components contributing to inter-accession differences. However, whether we apply Mantel Tests using genotype and volatile-based distance matrices, or fit models using the volatile-based measures as the response variable and the Structure-defined genetic clusters as predictors, we were unsuccessful in uncovering phylogenetic signal (see Results).

Our data highlight a central challenge in bridging the rapidly growing fields of population genomics and chemical ecology: the need to accomplish high-throughput, unbiased sampling of large populations with diverse chemical phenotypes and simultaneously reduce or eliminate non-biological sources of noise in the data sets [60], [61]. The small sample sizes typical of GC-MS studies, combined with analytical noise, dilutes the strength of clustering observed in MDS and PCA analyses (Fig. 2). Similar factors likely placed limits on the Mantel Tests we performed, which can suffer from low power [62], [63], as well as for the models we fit, for which we have a narrow range of categories to treat as predictors (Structure-defined genetic clusters) due to the few number of genotypes assigned to each category (as the number of genetic clusters increases there are fewer accessions per cluster).

The inability to detect phylogentic signal between our genetic and phenotypic data contrasts with the positive correlation between the growth-related phenotypic data and the yeast's topology that was observed by Liti et al. (2009). We investigated this further by asking if the accessions that were fast or slow growers in the Liti et al. (2009) study corresponded to the high and low emitters of volatiles in our data set (total scent, peak normalized). Again, we observed no correlation between our study and theirs (Grand mean +/− SEM for predicted fast strains = 6252.98+/−631/64 (n = 10); for predicted slow strains = 7177.11+/−1543.35 (n = 4), T = −0.674, DF = 12, 1 tailed p = 0.26), suggesting that growth on grape juice as a substrate may not parallel that observed on standard SC growth media. Additionally, experiments by Liti et al (2009) utilized considerably more yeast accessions and replicates than we were able to accommodate in this study. The sampling density, combined with their use of a highly precise chemostat environment, likely reduced non-biological variation in their data and provided increased power to discriminate inter-accession differences. In particular, additional control to our sampling design could be made by employing more sophisticated growth conditions that maintained constant cell densities across accessions, as well as a steady sugar source (grape juice). Though our simplified growth conditions are limited in their ability to remove all non-biologically relevant variation from the study, our analyses demonstrate that appreciable and consistent differences persist between strains across replicates.

### The volatile data and the genetic topology of the accessions are informative given what is known about the fly olfactory system

Despite the lack of correlation between our volatile data and the accessions genetic topology, the fact that the PCA and MDS analyses of volatile data are significantly structured among accessions remains informative for future behavioral experiments (Fig. 2). In particular, all compounds that are represented by the 3 significant PC loadings (Table 2) are known to be active ligands for *D. melanogaster*'s chemosensory receptor [36], [41], [43], [64], [65]. Furthermore, there is additional experimental evidence that relates several of the receptors for these compounds to behavior (Table 3). For example, recent work is beginning to associate polymorphism within olfactory receptors genes with differences in odor-mediated behavior, including OR22a in relation to ethyl esters [66]. Another olfactory receptor, OR42b, which detects ethyl acetate, has been shown to be within a neural circuit that underlies food-searching behavior in response to starvation [67]. And IR64a, which has been shown to detect isobutyric acid, is important for for avoidance response to acid [68]. That these varying yeast volatile compounds are central fruit fly attractants suggests that behavioral choice assays conducted with the most divergent yeast strains from our data set could be used to test how flexible fly attraction is to varying ratios of these key volatiles. Candidate pairs that would likely present insightful tests would include 1889 and 1888, both of which have been selected for fermentation purposes but which significantly differ in amounts of isobutyric acid and acetoin, or 2094 and 2106, both of which are wild accessions, but significantly differ in amounts of 2-phenylethanol as well as ethyl esters and 3-methyl butanol and 3-methyl butyl acetate. More complicated multi-accession preference experiments could be envisioned utilizing additional tools such as 4-arm olfactometers [69] or higher throughput preference/sensitivity assays (for example [70]).

Finally, the common garden design we utilized almost certainly underestimates the amount of metabolic volatile variation that these accessions are capable of generating. An obvious follow-up study would be to investigate the genotype by substrate interaction for these yeast accessions. Because they were isolated from discretely different substrates (Table 1), and that several were domesticated for particular anthropocentric aims (fermentation), it would not be surprising if artificial selection has optimized particular strains for the use of specific sugars or by-products (e.g. ethanol), and otherwise resulted in differentiation within the underlying metabolic networks. Such patterns are emerging from recent research on the unintended effects of artificial selection on floral volatile composition in domesticated snapdragon, rose and other flowers [71]. We expect that headspace samples collected from our yeast accessions grown on different substrates would provide additional variation for both the main components we detect here, but potentially also for novel compounds not observed when grape juice or sucrose solutions are used as substrates [58].

## Materials and Methods

### Yeast accessions and culture

Fourteen strains of *Saccharomyces cerevisiae* that had previously been isolated from around the world on diverse substrates (including rotting fruit, feces and alcoholic beverages) were included in this study (see Table 1). Different strains were assigned code numbers that revealed no information about their origins and were cultured blindly by JRA and CS (June–July 2010) in preparation for volatile collection and GC-MS analysis using a non-polar GC column (see below). The 14 strains were haphazardly assigned to two groups of seven and were cultured in a “common garden” as described below. Seven accessions with highly divergent volatile profiles were cultured using the same protocols and analyses were repeated on a polar GC column (see below) by JRA and Y-RL (Oct.–Nov. 2011).

For each yeast strain, colonies were grown on standard YPD plates overnight at 37°C. Colonies from the same plate were picked in the morning and combined into a stock sample of 950 ml of 1∶10 solution of grape juice (Cascadian Farm 100% juice) to water. The number of cells within the stock sample volume was estimated using a hemocytometer (Hausser Scientific), and was kept at approximately 10^6^. To grow small yeast samples for headspace sampling, 2 ml of well-mixed stock solution was placed into autoclaved 5 ml glass tubes and grown overnight (18 hrs) at 30°C in a shaker incubator (cf. 20 hrs. by Becher et al. 2012 [34]). This procedure controlled the number of cells at the start of each experiment, however because the vials were not subject to modification over their growth (as done with chemostat equipment), the cell count was not kept constant over the growth period.

### Volatile Collections

Volatiles were collected using micro thermal adsorbent traps constructed from cut glass capillary tubes (1.6 mm OD, 1.3 mm ID, 25 mm long) filled with 5 mg of Tenax TA (60/80 mesh size) absorbent (Supelco, Inc.) packed between plugs of silanized quartz wool (Restek, Inc.). This method was used in order to avoid co-elution of small, highly volatile fermentation products with the solvents (e.g. hexane, methylene chloride) typically used to desorb trapped volatiles (see [34]). Tenax traps were hand constructed, cleaned with methanol and oven dried at 50°C before use. Clean traps were attached to 9V battery-operated PAS-500 vacuum pumps (Spectrex, Inc.) using an adapter constructed from glass Pasteur pipettes, silicone tubing and Teflon tape. Pump flow rates were calibrated to 200 ml air/min each day using a bubble flow meter (Gilmont, Inc.) to standardize collection protocol across all replicates.

Yeast volatiles were sampled in a randomized block design each day, for each group of seven accessions, until at least five replicates for each accession had been collected. Sampling included an ambient control (empty glassware) and a negative control (non-inoculated grape juice substrate). Samples incubated prepared overnight as described above, and at 10:00 hrs the following morning, each vial was capped with a gasket of nalophan oven bagging (Toppits, Inc), placed into a water bath in a 10 ml glass beaker and moved to a hot plate at 30°C for volatile collection. A small hole was cut into each gasket, through which the Tenax trap could be inserted, with its associated vacuum pump suspended above the yeast culture using a vertical ring stand and clamps. Volatile samples were collected for 5 min, placed into labeled 1.5 ml amber glass autoinjector vials (National Scientific, Inc.) and stored at room temperature until GC-MS analysis.

### Chemical Analysis of Volatiles

Volatiles were collected (independently by CS and Y-RL) and peak areas were integrated (by RAR) blindly, without full knowledge of accession identity or history. Trapped volatiles were analyzed using direct thermal desorption (TD) coupled with gas chromatography-mass spectrometry (GC-MS), by placing Tenax traps into removable injection port liners and purging for two minutes with the split valve open to bleed ambient gas from the injection port (Optic 3 high performance GC injector, ATAS GL, International BV). Volatiles were desorbed from the Tenax traps using ballistic heating (from 30 to 200°C at 15°C/sec), through the Optic 3 external flow control system coupled with GC-MS. Desorbed volatiles were swept into a Shimadzu GC2010+ by a mobile phase of ultra-pure helium gas during a 1 min splitless injection, thereafter maintaining a constant column flow of 1 ml/min at a 20∶1 split ratio. Yeast volatiles were separated on a non-polar (RXI-5MS [5% diphenyl/95% dimethyl polysiloxane, comparable to DB-5], 30 m, 0.25 mm ID, 0.25 m film thickness; Restek, Inc) GC column using a temperature program increasing from 30°C (after a 2 min hold) at 10°C/min to 275°C, then holding for 6.5 min. A second round of analyses were conducted using a polar (Stabilwax [polyethylene glycol, comparable to DB-wax], 30 m, 0.25 mm ID, 0.25 m film thickness; Restek, Inc.) GC column, held 1 min at 30°C, increased 6°C/min until 105°C, then heated at 15°C/min to 230°C and held for 9.17 min. The GC was coupled to an electron-impact, quadrupole MS with an EI scan sensitivity of 1 pg octafluoronaphthalene (signal: noise >200) in single ion mode (SIM, m/z = 272). Eluting volatiles were subjected to 70 eV of ionizing energy and were scanned at 5 scans per sec from m/z 40–350 daltons. N-alkane standard compounds were injected under the same chromatographic conditions in order to render all retention times as Kovats indices [72]. Identification of unknowns proceeded by comparison with mass spectral libraries [73], co-injection with authentic standards, and comparison with published volatile data for bakers yeast [34], [57].

For each day's yeast samples, we determined which GC peaks were legitimate yeast fermentation products by overlaying chromatograms with those of ambient and grape juice controls and subtracting common peaks. In particular, grape juice headspace contained small amounts of many straight chain organic acids, so we omitted these acids from consideration as yeast volatiles unless their peak areas were 4× greater than those present in controls (e.g. acetic acid). For all volatiles specific to yeast, GC peaks were hand-integrated using GC-MS Solutions 1.2a software (Shimadzu Scientific Instruments, Inc.). Because some compounds were present at the threshold of detection in total ion chromatograms (TIC), we searched below baseline using single ion mode (SIM) by identifying m/z 91 and 104 (phenylethyl derivatives), m/z 88 (aliphatic ethyl esters), m/z 70 (aliphatic esters of 3methylbutanol) m/z 73 and 60 (aliphatic acids) at 100× sensitivity. Thus, volatile compounds scored as absent from specific samples were not detected using these methods. It is not straightforward to add internal standards to samples collected by TD-GC-MS, especially when the volatiles are being emitted from a fluid matrix through fermentation. Instead, we reasoned that an appropriate internal standard would be a volatile present in grape juice that was unlikely to be metabolized by yeast. For these purposes, the most reliable compound present in grape juice was the definitive flavor component methyl anthranilate [55], [74]. We normalized all integrated yeast volatile peaks in each sample by dividing their peak areas by that of methyl anthranilate in the same sample, to control for unintended variation in trap capacity and the injection-desorption process. These normalized peak areas represent the raw chromatographic data used in subsequent statistical analyses. Replicates in which large peaks of glycerol were detected were omitted from analysis, as yeast is known to ferment ethanol into glycerol [75]. We interpreted these cases to represent replicates in which fermentation had proceeded too far.

Mass spectra and chromatographic data have been archived digitally at the eCommons site at Cornell University (http://ecommons.library.cornell.edu).

### GC-MS Data extraction and Statistical Analysis

Once volatiles were confidently attributed to yeast (not to grape juice or ambient contaminants) and were identified to the fullest extent possible, two multivariate methods were used to explore accession-related variation in volatile composition. The first method, Principal Components Analysis (PCA), was used to identify correlated quantitative variation amongst the 8 volatile compounds that were detected consistently in all accessions and replicates using our TD-GC-MS protocols. These compounds were 3-OH-2-butanone ( = acetoin), 3-methyl butanol, isobutyric acid, 3-methyl butyl acetate, 2-phenylethanol, and the esters ethyl-hexanoate, -octanoate and -decanoate. Ethanol, acetic acid and 2,3-butanediol were omitted from this PCA because of poor resolution on the non-polar GC column, due to overshadowing by the dominant peak of 3-methyl butanol, which constituted 87–95% of all yeast volatile emissions by peak area (Table S2). We ordinated a correlation matrix of normalized, untransformed GC-MS peak areas for each volatile compound over 4–7 replicates of all 14 accessions, using varimax rotation, identifying as principal components all factors with eigenvalues greater than or equal to unity (SPSS 11.5 for Windows).

The second method, Multidimensional Scaling (MDS), was used to explore variation in the chemical composition of volatile blends including compounds not present in all samples, to determine whether rare or unique volatiles might contribute to accession-specific novel blends. To perform MDS with our data set, we used the PRIMER v6 program [76]. Normalized peak area data for all detected compounds were square root transformed [77] and then were used to calculate a Bray–Curtis similarity index and an associated stress value, such that stress values approaching zero indicate a closer fit between the reproduced ordination and the observed matrix (Clarke 1993). The significance of differences in scent composition between yeast accessions was measured using analysis of similarity (ANOSIM; [76]. ANOSIM is a multivariate procedure analogous to ANOVA, which calculates the test statistic R (ranging from 0 to 1) and its p-value. R is a relative measure of separation between groups; an R-value of zero indicates random grouping, whereas values closer to unity indicate that samples within defined groups are more similar to each other than to those of different groups [76]. The significance of the R statistic was determined by 10,000 random permutations of the grouping vector to generate an empirical distribution of R under the null model. A significant R value justifies further exploration of group-level differences, as visualized by plotting Bray-Curtis ordination coefficients through MDS. When samples cluster to some extent with pre-defined groups, it is appropriate to explore the contributions of specific compounds to such patterns using a post-hoc test of contributions to similarity (SIMPER) [78]. To indicate associations between specific volatile compounds and different coordinates in 3 dimensional Cartesian scent space via MDS, we identified the accessions with the largest and smallest mean coefficients for each dimension, then determined via SIMPER which compounds largely explained their differences (see Table S2). Thus, we were able to assign chemical landmarks within MDS as one might associate specific principal components with factor loadings for such compounds [77].

### Genomic Data Collection

Genome-wide SNP datasets exist for 11 of the 14 of the yeast accessions with which we carried out the above chemical phenotyping (1885, 1902, 1889, 1897, 1911, 1903, 1888, 1893, 2093, 2097 and the reference genome 1876). These resources were generated by two separate efforts (Liti et al. 2009; Schacherer et al. 2009). The Liti et al. resequencing effort used primarily ABI 370 and Illumina sequencing technologies. The Schacherer effort used tilling microarrays to identify polymorphisms.

Nine of the 11 yeast accessions used in our analyses were analyzed by Liti et al (2009), and the remaining two (2093, 2097) by Schacherer et al. (2009). Previously, Schacherer et al. (2009) compared their SNP calls with those of Liti et al. (2009) and estimated a low false-positive rate (92% correspondence between SNPs called between platforms), and high coverage when comparing SNP calls with quality scores >30 (a median of 72% of the SNPs per strain). Based on these measures, and the fact that only two strains exist within the data set of Schacherer et al. (2009), we reasoned that for our particular analyses intersecting data for all 11 accessions would be little effected by potential ascertainment biases.

Data sets were combined by first creating SNP tables for the Liti et al. (2009) data, and then extracting the overlapping SNPs from the set of Schacherer et al. (2009). To do this we used the “alicat.pl” utility [79] to output only polymorphic sites from the 9 overlapping accessions, and thresholded on Phred Quality Scores >30. Liti et al. (2009) employed an imputation approach to infer missing data, and it is from these data sets that we extracted the SNPs for our study [45], [79]. We further filtered our SNP table based on per-site missing data, discarding all sites that had >4 accessions without a base called, as well as requiring that at each site the subset of 9 samples had polymorphism segregating within it. In total, this filtered dataset had 112,188 SNPs across the 16 chromosomes.

The coordinates of the SNPs were initially outputted relative to the complete alignment of the genomes sequenced by Liti et al (2009). To ensure that we converted these coordinates to the correct reference position, we BLASTed [80] (v2.2.26) a chromosome segment of >150 bp centered on the SNPs against the reference genome using BLAST's default settings. Only SNPs with unambiguous positions were analyzed in this study. Based on the reference SNP positions, the orthologous bases from the two additional accessions in the Schacherer et al. (2009) study were extracted using their “all_SNPs.txt” file, converting the binary coding in this file into nucleotides based on the segregating bases observed in the first 9 strains. We manually checked bases and alignments to ensure that they were coherent across position labels. After combining datasets, we had a total of 5,227 intersecting SNPs across the 16 chromosomes (File S1).

### Neighbor-joining tree construction

A fasta file was written from the intersecting SNP table generated from above. SplitsTree [81] (v4.12.6) was used to generate the Neighbor Joining tree and to compute the bootstrap values (n = 10000) for Fig. 1. Additional grouping annotations on the tree were added subsequently according to the Table S2 published by Liti et al (2009) and S1 in Schacherer et al. (2009).

### Population Structure Analysis

Structure [82], [83] (v2.3.4) was used to infer the number of genetic clusters within our data set of 5,227 SNPs across the 11 accessions. The linkage model (Falush et al 2003) was used over values of K = 1,2,3,4,5,6. Each run at these 6 values was carried out in triplicate to ensure consistent results, using 70,000 iterations (the first 25,000 used as burn-ins). Investigation of MCMC convergence was accomplished through plots generated by Structure's front end. The ancestry estimate plots (Fig. 1) were generated using distruct1.1 [84].

### Tests of Correlation Between Volatile and Genetic Data

We tested for correlation between the clustering observed within our yeast volatile data and the clustering of the genomic SNP data by carrying out Mantel tests and regression testing. The first type of Mantel test was carried out between a Euclidean distance matrix constructed from the centroid MDS values for the non-polar GC-MS experiment accessions and a Euclidean genetic distance matrix generated from the yeast SNP data. Significance was determined using 10,000 permutations. The second type of Mantel test utilized our replicate experiments in order to propagate error, and resulted in a distribution of p-values against which to compare our empirical value. To do this, Euclidean distances matrices between MDS points were “regenerated” by randomly filling each cell by sampling from the same cell across our replicate data. At each iteration, the regenerated matrix was correlated to the fixed genetic distance matrix. For each individual test, significance was determined using 1000 permutations, and a total of 5000 iterations were carried out. The “mantel.rtest” function within R's [85] ade4 library [86] were used.

The initial regression approach was to treat the genetic clusters that were inferred from Structure (above) as categorical response variables and ask if the clustering of the chemical data was predictive of these genetic categories. Restricting the number of Structure-defined clusters to three (K = 3; an attempt to maintain the largest number of yeast accessions per cluster as possible while simultaneously not allowing for arbitrary assignment to genetic clusters when K = 2 and inferred ancestry is close to 50/50), we fit categorical logistic regression using PC's as predictors, with and without an interaction term. Model fitting was carried out using the R package VGAM (using the Multinomial logit model, “family = multinomial”; [87], [88]). To further accommodate the K = 2 Structure partition (above), we carried out a linear regression with the matrix of proportions of ancestry (inferred by Structure) as the response variables and the chemical PC's as the predictor variables. The linear regression was carried out in R using the “lm” function in the Stats package [85].

## Supporting Information

File S1 **Fasta file for overlapping sites mined form the data sets of Liti et a. 2009 & Schacherer et al. 2009.**
(FASTA)

Figure S1 **Resulting posterior probabilities for the number of genetic clusters (K) the dataset inferred by Structure (v2.3.4; Pritchard et al., 2000; Falush et al., 2003).** Each of the three colored lines indicates an independent run of the inference procedure. The maximum for the three runs is located at K = 5.
(PDF)

Table S1 **Yeast accession, sample size, mean and standard error of mean (SEM) relative percentage of total ion current, normalized GC-MS peak areas for each volatile compound identified.**
(XLS)

Table S2 **SIMPER analysis of Bray-Curtis chemical similarity within and between 14 yeast accessions based on GC-MS analysis on non-polar column.**
(XLS)
