# Supplementary figures and images for: Can Yeast (S. cerevisiae) Metabolic Volatiles Provide Polymorphic Signaling?
Source: PLoS One. 2013 Aug 19;8(8):e70219. doi: 10.1371/journal.pone.0070219 (PMC3747187; doi:10.1371/journal.pone.0070219)

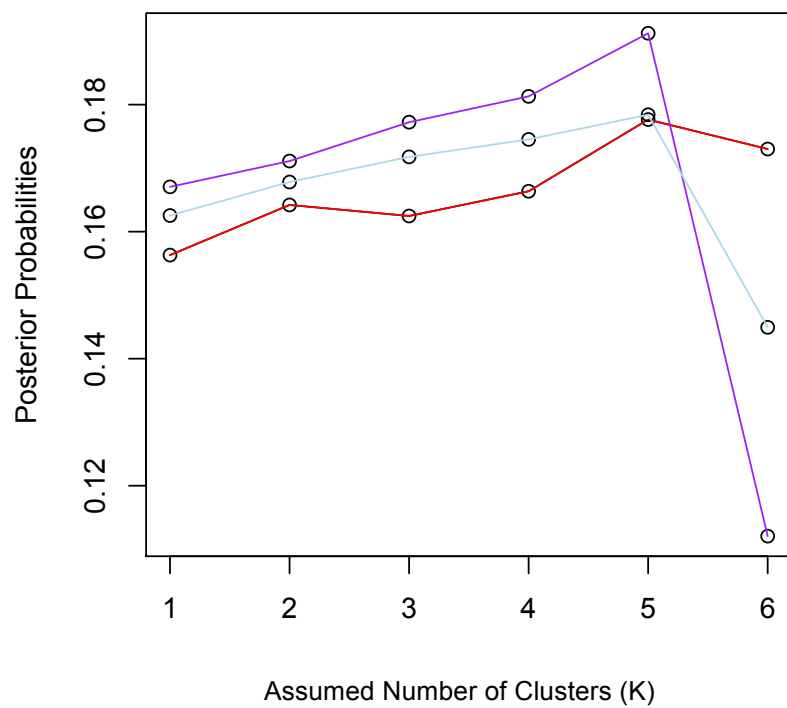

Supplement: Figure S1 — Resulting posterior probabilities for the number of genetic clusters (K) the dataset inferred by Structure (v2.3.4; Pritchard et al., 2000; Falush et al., 2003). Each of the three colored lines indicates an independent run of the inference procedure. The maximum for the three runs is located at K = 5. (PDF) [file pone.0070219.s002.pdf]
